# Identification and Characterization of a QTL for Growth of *Fusarium circinatum* on Pine-Based Medium

**DOI:** 10.3390/jof8111214

**Published:** 2022-11-16

**Authors:** Benedicta S. Swalarsk-Parry, Emma T. Steenkamp, Stephanie van Wyk, Quentin C. Santana, Magriet A. van der Nest, Almuth Hammerbacher, Brenda D. Wingfield, Lieschen De Vos

**Affiliations:** 1 Department of Biochemistry, Genetics and Microbiology, Forestry and Agricultural Biotechnology Institute (FABI), University of Pretoria, Pretoria 0002, South Africa; 2 Agricultural Research Council, Biotechnology Platform, 100 Old Soutpan Road, Onderstepoort, Pretoria 0002, South Africa; 3 Department of Zoology and Entomology, Forestry and Agricultural Biotechnology Institute (FABI), University of Pretoria, Pretoria 0002, South Africa

**Keywords:** QTL, *Fusarium circinatum*, genetic linkage map, *Fusarium temperatum*

## Abstract

*Fusarium circinatum* is an economically important pathogen of pine and resides in the *Fusarium fujikuroi* species complex. Here we investigated the molecular processes underlying growth in *F. circinatum* by exploring the association between growth and the nutritional environment provided by the pine host. For this purpose, we subjected a mapping population consisting of *F. circinatum* X *F. temperatum* hybrid progeny to an analysis of growth rate on a pine-tissue derived medium. These data, together with the available genetic linkage map for *F. circinatum*, were then used to identify Quantitative Trait Loci (QTLs) associated with growth. The single significant QTL identified was then characterized using the available genome sequences for the hybrid progeny’s parental isolates. This revealed that the QTL localized to two non-homologous regions in the *F. circinatum* and *F. temperatum* genomes. For one of these, the *F. circinatum* parent contained a two-gene deletion relative to the *F. temperatum* parent. For the other region, the two parental isolates encoded different protein products. Analysis of repeats, G+C content, and repeat-induced point (RIP) mutations further suggested a retrotransposon origin for the two-gene deletion in *F. circinatum*. Nevertheless, subsequent genome and PCR-based analyses showed that both regions were similarly polymorphic within a collection of diverse *F. circinatum*. However, we observed no clear correlation between the respective polymorphism patterns and growth rate in culture. These findings support the notion that growth is a complex multilocus trait and raise the possibility that the identified QTL contains multiple small-effect QTLs, of which some might be dependent on the genetic backgrounds. This study improved our current knowledge of the genetic determinants of vegetative growth in *F. circinatum* and provided an important foundation for determining the genes and processes underpinning its ability to colonize its host environment.

## 1. Introduction

The genus *Fusarium* is undoubtedly one of the most important fungal genera [1,2]. This is especially true for the *Fusarium fujikuroi* species complex (FFSC) [3], which includes pathogens that are toxigenic to humans and animals [4,5,6,7] and that can cause significant losses to various agricultural and forestry crops [8,9,10,11]. Their socioeconomic importance is also reflected by the large number of FFSC species for which whole-genome sequences are currently available in the public domain. Indeed, data for at least 50 FFSC species are available in the genome database of the National Center for Biotechnology Information (NCBI; https://www.ncbi.nlm.nih.gov (accessed on 30 June 2022)). Some of these include the maize pathogen *Fusarium verticillioides* [12], the rice pathogen *Fusarium fujikuroi* [13], and the pine pitch canker pathogen *Fusarium circinatum* [14]. The availability of these resources, combined with the increasing affordability of whole-genome sequencing [15], continues to present opportunities for studying and understanding the molecular basis underlying phenotypic traits of FFSC fungi.

The genomes of species in the FFSC are characterized by high levels of macrosynteny [13]. The corresponding chromosomes of related FFSC species remained homologous across individual molecules and were characterized by a high degree of shared synteny and co-linearity [16]. This macrosynteny is commonly observed for chromosomes that evolve predominantly through homologous recombination [17]. The macrosyntenic nature of FFSC genomes was thus consistent with their relatively recent emergence during the Late Miocene period [18], and evolutionary trajectories reflect high levels of hybridization and introgression during older [19,20] and more contemporary time scales [21,22,23]. 

Macrosynteny among interfertile species is of value to investigate the molecular basis of specific phenotypic traits. Interfertility facilitates the construction of genetic linkage maps (GLMs) that are used to identify quantitative trait loci (QTLs) underlying traits of interest [24,25]. Previous work on *F. circinatum* QTL analyses was performed on laboratory-derived hybrid offspring of *F. circinatum* and its close relative, *F. temperatum* [26], which were later used to identify a QTL underpinning morphology of colony margins and growth at 25 °C and 30 °C [27]. This information was complemented by whole genome macrosynteny analyses [16]. Moreover, the *F. circinatum* growth QTL harbored a ca. 12 Kilobase pair (Kbp) region that was unique to the sub-telomeric region of chromosome three of the fungus [28]. 

Fungal growth is a complex trait controlled by multiple loci, which can be influenced by numerous environmental factors [29]. These include abiotic (e.g., temperature, nutrient availability, pH, water availability, etc.) and biotic factors (e.g., presence of other competitors, mutualists, etc.) [30,31,32,33,34]. For these factors, the growth response was governed by single or multiple loci, which could be unique to a particular fungus or common among various fungi. The ca. 12 Kbp QTL associated with growth in *F. circinatum* is a species-specific locus directing growth. Two notable examples of genes that affect growth include transcription factors, the HAP (heme activator protein) complex, and Ras2 (rat sarcoma 2). The HAP complex regulates the transcription of diverse biological processes [35,36,37], while Ras2 regulates signaling pathways [38]. These findings were supported by deletion mutational analyses, which resulted in stunted growth [39,40,41]. 

The overall goal of this study was to extend our knowledge of molecular processes underlying growth in *F. circinatum* by exploring the possible association between growth and the nutritional environment provided by the pine host. We utilized the *F. circinatum* X *F. temperatum* hybrid progeny, and GLMs generated previously [26]. *F. circinatum* can also colonize a wide variety of non-pine hosts asymptomatically [42,43,44,45,46], including the asymptomatic endophytic infection of maize [43,47]. Within the tissues of their respective pine and maize hosts, *F. circinatum* and *F. temperatum* require different nutrients and chemical compounds. Therefore, the first of our four aims was to assay the vegetative growth of the *F. circinatum* X *F. temperatum* hybrid progeny on a pine-based medium. Secondly, we used these growth data to localize the relevant QTLs on the *F. circinatum* GLM. Thirdly, we used genome comparisons to provide a detailed characterization of the identified QTLs. Lastly, we determined whether the growth patterns associated with these loci could also be observed in *F. circinatum* isolates collected in natural and planted pine stands. The results of this study allow for an improved understanding of the genetic determinants of vegetative growth in these fungi and the biology and ecology of *F. circinatum*.

## 2. Materials and Methods

### 2.1. Fungal Isolates and Growth Medium

This study used two sets of isolates. The first set included the interfertile fungal isolates *F. circinatum* (CMWF 350; FSP34) and *F. temperatum* (CMWF 389; Netza9) and their 94 F_1_ hybrid progeny (Supplementary File S4, Table S1) that were previously used for GLM construction [26]. The second set included 19 genetically diverse *F. circinatum* isolates that were collected from various locations in South Africa, Mexico, California, and Florida during previous studies (Table 1). 

Isolates were grown on one of two types of growth media, potato dextrose agar (PDA; 15 g/L; Biolab) and pine-tissue-derived agar medium. The latter was prepared by chopping the above-ground parts of 6-month-old *Pinus patula* seedlings into small pieces, of which 40 g was added to 1 L of H_2_O and autoclaved at 120 °C for 30 min. The extract was filtered through Whatman Grade 1 filter paper (GE Healthcare Life Sciences) to remove the plant debris. The filtrate was mixed with malt extract (20 g/L; BD Difco^TM^) and agar (10 g/L; BD Difco^TM^), autoclaved, and poured into plates. The medium is referred to as PMEA (for pine-tissue extract containing malt extract agar), and Supplementary File S1 provides an overview of the metabolites it contains, as determined according to Hammerbacher et al. [52] and Gonzalez-Cabanelas et al. [53].

### 2.2. Vegetative Growth Assays

Both sets of fungi included in this study were grown on PDA for 7 days at 25 °C in the dark. Mycelial plugs from the colony margins (using a sterile 5 mm cork borer) were then used to inoculate 90 mm Petri plates containing PMEA. For both sets of fungi, 10 plates per isolate were inoculated, followed by incubation in the dark at 25 °C for 7 days. The colony diameter was then recorded by taking the mean of two perpendicular measurements per plate.

The results of the two growth experiments were subjected to various statistical analyses. In the case of the hybrid progeny and their parents, Student *t*-tests were used to identify isolates that grew significantly faster than the parental isolates. This dataset was also used to determine broad-sense heritability (H^2^ = σ^2^_G_/σ^2^_P,_ i.e., the proportion of genotypic to phenotypic variance) using single-factor analysis of variance (ANOVA) to determine the effect of the environment on the observed trait. The growth measurements obtained for the set of 19 isolates were subjected to ANOVA, after which means were compared using Tukey’s honest significance test (HSD) performed using R statistical software V4.1.0 (R Foundation for Statistical Computing, Vienna, Austria).

### 2.3. QTL Identification and Genomic Localization 

MapQTL 6 [54] was used to perform quantitative trait analysis using the GLM data generated by De Vos et al. [26]. In order to analyze the results from the haploid cross-population, the HAP1 population code was utilized. The Kruskal-Wallis test was used to link the studied trait to the markers. Interval mapping using 1000 permutations at 1 centiMorgan (cM) intervals was carried out to determine significant QTLs on the GLM using the Log-of-Odds (LOD) values. Afterward, the multiple-QTL Model (MQM) procedure was used to identify QTLs with a significance threshold value of *p* < 0.05, *p* < 0.01, and *p* < 0.001. 

Identified QTLs were localized to the genome of *F. circinatum* FSP34 [55] using the genomic locations of the AFLP markers [26], flanking them, and the methods described by De Vos et al. [16]. Briefly, this involved estimating the position of a QTL relative to the AFLP markers flanking it based on the known distance (in bp and cM) between the markers. Once localized, QTL regions in the *F. circinatum* FSP34 genome [55] were compared to homologous regions in the genome of *F. temperatum* Netza9 [56] using MUMmer v. 3.22 [57]. The latter employs suffix trees for identifying matching regions in two DNAs that can serve as anchors during the alignment process. For comparative purposes, the QTL regions in the FSP34 genome were also investigated in the genome of strain KS17 of *F. circinatum* [28] using MUMmer. 

### 2.4. Characterization of Genes in QTL Regions 

Sequence regions corresponding to QTLs were extracted from the genomes of *F. temperatum* Netza9, and *F. circinatum* strains FSP34 and KS17 and subjected to gene content analysis. This involved gene prediction using WebAUGUSTUS [58] with *Fusarium graminearium* gene models and confirmation of gene intron/exon boundaries using available mRNA data for *F. circinatum* FSP34 [59]. InterProScan 5 [60,61] was used for identifying protein functional domains, gene ontology, and protein family membership.

Additionally, the presence/absence of genes predicted to occur in QTL regions were verified using PCR and sequencing. For this purpose, we included *F. circinatum* (FSP34 and KS17) and *F. temperatum*, as well as the 19 other isolates of *F. circinatum.* DNA was extracted from 7-day-old PDA cultures using the method of Doyle & Doyle [62] with modifications as made by Ashktorab & Cohen [63]. Suitable primers (Supplementary File S2) were designed using PRIMER3 version 4.1.0 [64], and PCR was performed using KapaTaq^TM^ (Kapa Biosystems, Wilmington, MA, USA) or LongAmp^®^ Taq (New England BioLabs, Ipswich, MA, USA) using the manufacturer’s instructions. Amplicons were purified with G50 Sephadex (Sigma, Steinheim, Germany) and sequenced using the ABI3500x1 Genetic Analyzer (Applied Biosystems, Foster City, CA, USA) based at the University of Pretoria (South Africa).

### 2.5. G+C, Repeats, and Transposable Elements in the QTL Region

The QTL regions in *F. circinatum* FSP34 were further analyzed for G+C content using CLC Genomics Workbench v21.0.2 (Aarhus, Denmark), as well as repeat and transposable elements (TEs) content using REPET v2.5 [65]. For the complete TEs detected, BLAST searches were used to determine their presence in other locations within the genomes of *F. circinatum* (FSP34 and KS17) and *F. temperatum*. We also subjected the extracted QTL regions to fine-scale RIP analysis with the RIPper [66] (https://theripper.hawk.rock (accessed on 25 February 2022)) using standard parameters (1000 bp window and 500 bp step size). As suggested by van Wyk et al. [66], sequence windows with the following values were regarded as RIP-affected or RIP-positive: Product Index Value: 0.8 ≤ x > 1.1; Substrate Index Value: x ≤ 0.9 and Composite Index Value: x > 0.

### 2.6. Genome Assembly Confirmation

In order to ensure that the results were not artifacts inherent to genome assemblies [67], the sequence in QTL regions was verified for *F. circinatum* FSP34. This was conducted by mapping the raw sequence reads of *F. circinatum* FSP34 back to the genomes of both *F. circinatum* FSP34 and KS17, as well as *F. temperatum*, using CLC Genomics Workbench v21.0.2 (Aarhus, Denmark). The sequence reads consisted of long reads from a MinION sequencer (Oxford Nanopore Technologies, Oxford Science Park, Oxford, United Kingdom) and a 550-bp paired-end library using the Illumina HiSeq 2500 (Macrogen, Seoul, Korea) [55]. We also attempted to PCR amplify across any predicted indels in the QTL regions, using primers designed with PRIMER3 and as described above (Supplementary File S2). 

## 3. Results

### 3.1. A Major QTL Determines the Growth Rate of F. circinatum on Pine-Tissue Derived Medium 

**Figure 1:**
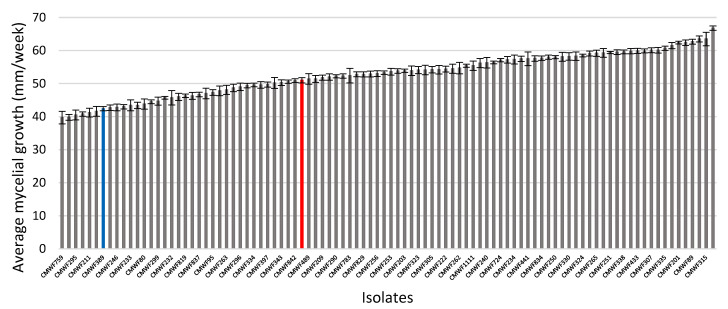
Mycelial growth differences between *F. circinatum* FSP34 (**red**), *F. temperatum* (**blue**), and the F_1_ progeny were observed after 7-day growth on PMEA (pine-tissue extract containing malt extract agar) growth medium. The error bars represent the standard deviation.

The *F. circinatum* isolate (FSP34; indicated in red in Figure 1) grew significantly faster (*p* = 2.77 × 10^−7^) on pine tissue-derived medium than the maize/teosinte pathogen *F. temperatum* (CMW389; indicated in blue in Figure 1, Table in Supplementary File S1). After 7 days of incubation, FSP34 produced average colony diameters of 51 mm as opposed to 43 mm for *F. temperatum*. Analysis of their 94 F_1_ progeny (indicated in grey in Figure 1) revealed that 51 and 81 of the strains grew significantly faster (*p* < 0.05) than their respective *F. circinatum* FSP34 and *F. temperatum* parents. The broad-sense heritability of the trait tested was estimated at 0.98, which is indicative of the minimal influence of environmental variation on the mycelial growth of *F. circinatum* FSP34 and *F. temperatum* on pine-tissue derived medium. 

**Figure 2:**
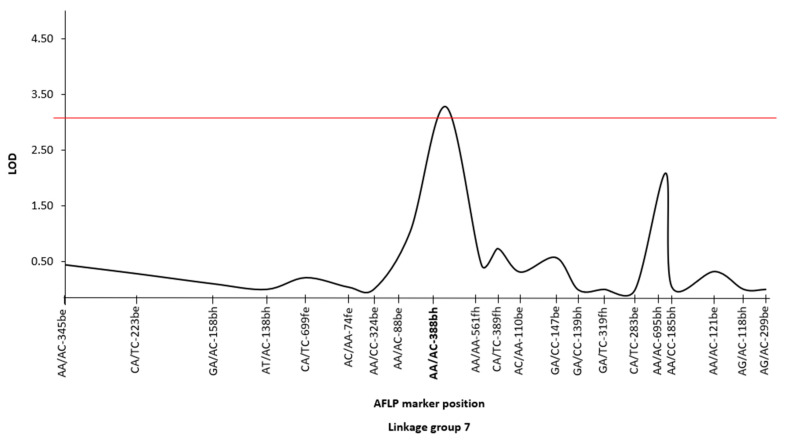
QTL was detected using MapQTL 6 [54] for the growth of *F. circinatum* FSP34 on a pine-based medium. The identified QTL (*p* = 0.05, indicated by the red line) was located in linkage group 7 (chromosome 11), with the nearest AFLP marker AA/AC-388bh being 4.5 cM away [26].

Quantitative trait analysis using the recorded growth measurements and the GLM data produced by De Vos et al. [26] allowed for the detection of one significant (*p* < 0.05) QTL that accounted for 16.4% of the total phenotypic variation for this trait (Figure 2). The QTL was localized to linkage group 7, where it was positioned closest to AFLP marker AA/AC-388bh. It was flanked by two other markers (i.e., AA/AA-561fh and AA/AC-88be), with the entire region encompassing 11.9 cM. As demonstrated previously [16], linkage group 7 corresponded to chromosome 11, where the size of the region flanked by markers AA/AA-561fh and AA/AC-88be was ca. 126 Kbp in the available genome assemblies of *F. circinatum* FSP34 and *F. temperatum*.

### 3.2. Comparison and Characterization of Genes in the Identified Growth QTL Region

**Figure 3:**
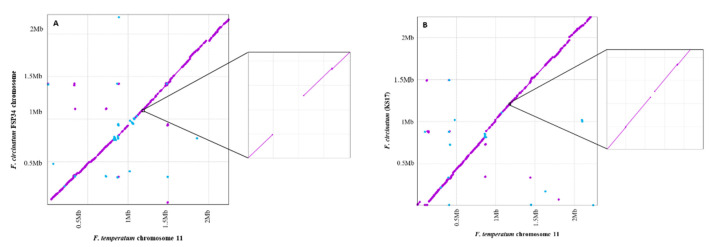

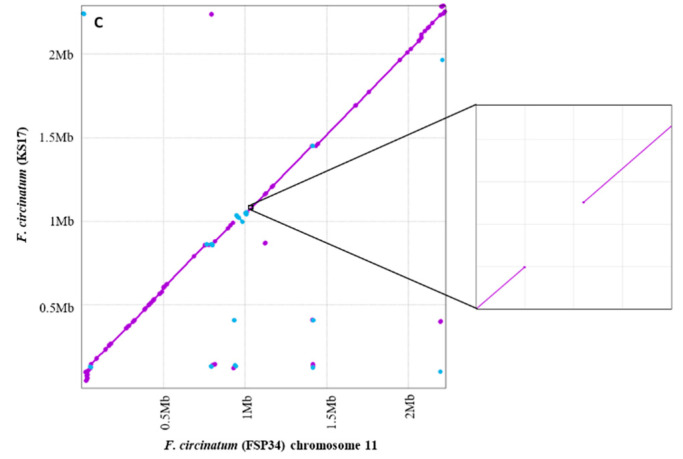
MUMmer-based comparisons of the region of chromosome 11 containing the identified QTL; (**A**) *F. circinatum* FSP34 versus *F. temperatum*, (**B**) *F. circinatum* KS17 versus *F. temperatum*, and (**C**) *F. circinatum* KS17 versus *F. circinatum* FSP34. In these dot plots, forward matches are indicated with purple dots, and reverse matches with blue dots. In all three cases, the insert shows a magnification of the target region, with a gap in synteny of around 7 Kbp.

Sequence comparisons of the QTL region revealed a large indel in the two fungal genomes examined. This was clear from our MUMmer-based alignments of chromosome 11, which showed an interruption in homology in the region containing the QTL (Figure 3). This was also evident when the *F. circinatum* FSP34 sequence was compared to another strain of *F. circinatum* (i.e., KS17). The *F. circinatum* FSP34 sequence thus appeared to harbor a large indel, not shared by *F. temperatum* and *F. circinatum* isolate KS17.

In order to ensure that this alignment break observed in the MUMmer plots was not due to genome assembly error, the raw reads for FSP34 [14,59] were mapped to the genomes of *F. temperatum*, *F. circinatum* FSP34, and *F. circinatum* KS17. Here, significantly fewer reads (*p* < 0. 05) mapped back to the QTL region of *F. temperatum* and *F. circinatum* KS17, and particularly also to the 7 Kbp region in *F. circinatum* FSP34. Reads that did map to this region were mostly single Illumina reads and not paired-end Illumina reads (Supplementary File S3), which suggested the region in *F. circinatum* FSP34 is repeat-rich [67,68]. The *F. circinatum* FSP34 MinION reads mapped to this indel region, but none mapped to the region in *F. temperatum* and *F. circinatum* KS17. The mapped reads in the indel region of *F. circinatum* FSP34 were found to be AT-rich across the 7 Kbp region. It is thus unlikely that the observed break in homology was an assembly artifact in the *F. circinatum* FSP34 genome. 

**Figure 4:**
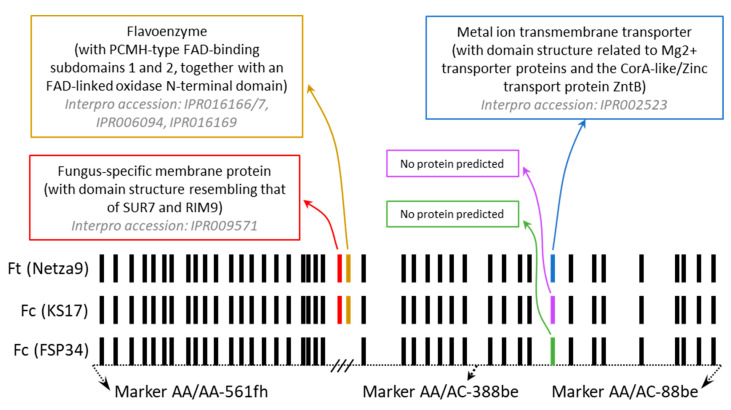
Schematic diagram of the organization of genes in the QTL region identified in the genomes of *F. circinatum* and *F. temperatum*. The two *F. circinatum* strains are indicated with Fc (FSP34) and Fc (KS17), while *F. temperatum* is indicated with Ft (Netza9). The black blocks represent the genes found to encode the same proteins in all three genomes. Colored blocks indicate non-homologous genes in the three genomes, where red indicates genes g13407 of *F. temperatum* and g13263 of *F. circinatum* KS17 and brown shows g13408 of *F. temperatum* and g13264 of *F. circinatum* KS17. Green, blue and purple, respectively indicate g13376 in *F. circinatum* FSP34, g13420 in *F. temperatum*, and g13276 in *F. circinatum* KS17. The three dashed lines indicate that the exact size of the region lacking the two genes in *F. circinatum* FSP34 is not known due to the presence of large retrotransposon Derivative (LARD) transposable elements.

Gene content analysis of the region flanked by AFLP markers AA/AA-561fh and AA/AC-88be showed that the *F. temperatum* and *F. circinatum* KS17 sequences contained two genes more than that of *F. circinatum* FSP34 (Figure 4). The QTL region of *F. circinatum* FSP34 encoded a total of 41 genes, while the corresponding sequence from *F. temperatum* had 43 genes (Table S2 in Supplementary File S4). Within the *F. circinatum* FSP34 sequence, AFLP marker AA/AC-388bh (i.e., the one located closest to the QTL) was positioned downstream of gene g13371. Analysis of the region in the genome of strain KS17 of *F. circinatum* [28] showed that it also encoded the 43 genes as in *F. temperatum*. Likewise, this was true for other sequenced members of the FFSC (i.e., *F. verticillioides*, *F. subglutinans*, *F. fujikuroi*, *F. pilosicola*, and *F. fracticaudum*) [12,13,69,70,71] that also contained the 43 genes in the region corresponding to those in *F. temperatum* (results not shown). 

The two genes missing in *F. circinatum* FSP34 corresponded to the 7 Kbp region identified using the read-mapping experiment mentioned above. In the assembled genome of this strain, it spanned 7090 base pairs (bp) that started directly after gene g13364 and ended immediately before gene g13365 (Table S3 in Supplementary File S4). In the genomes of *F. circinatum* KS17 and *F. temperatum*, these genes encoded a SUR7/RIM9-like membrane protein (genes g13407 of *F. temperatum* and g13263 of *F. circinatum* KS17) and a FAD-type 2 binding domain-containing protein (genes g13408 of *F. temperatum* and g13264 of *F. circinatum* KS17).

Apart from these two missing genes, 40 of the remaining 41 genes were similar between *F. circinatum* FSP34 and *F. temperatum* (i.e., they were homologous to their corresponding counterparts in the two fungi). They encoded products involved in diverse processes ranging from gene regulation to the transport of amino acids, inositol, and sodium solutes (Tables S3 and S4 in Supplementary File S4). The dissimilar gene in FSP34 (g13376) was located 36 Kbp downstream from gene g13365 and 17 Kbp downstream of gene g13371 in the estimated position of the QTL. In FSP34, it encoded a product that is different from that of the gene located at the same position in the genome of *F. temperatum* (i.e., g13420) (Figure 4). Yet, another product was predicted for the gene occurring at this position in the genome of *F. circinatum* KS17 (i.e., gene g13276). In the case of strain FSP34’s gene g13376 and strain KS17’s gene g13276. However, no functions could be predicted, while g13420 of *F. temperatum* encoded a putative magnesium transporter protein. 

### 3.3. G+C, Repeats, TEs, and RIP in the QTL Region

The G+C content of the genomic region containing the QTL differed between the respective fungi examined. The region in *F. circinatum* FSP34 had a G+C content of 19.40%, whereas the corresponding regions in *F. circinatum* KS17 and *F. temperatum* were respectively characterized by 35.19% and 46.92% G+C. Except for the latter value, these G+C content estimates were substantially lower than what has been reported as genome-wide averages. The average G+C content of the entire *F. circinatum* FSP34 genome is 47.40% [14], *F. circinatum* KS17 is 44.69% [28], and *F. temperatum* is 47.00% [14,56]. 

**Figure 5:**
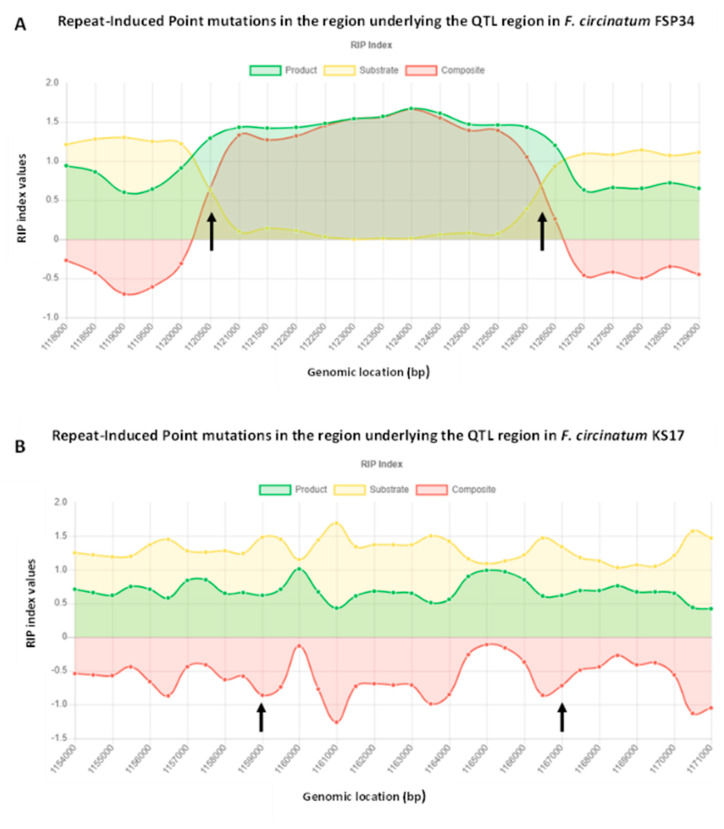
Graphs showing evidence of Repeat-Induced Point (RIP) mutations in the region underlying the QTL region of (**A**) *F. circinatum* FSP34 (indicated by the arrows) and (**B**) *F. circinatum* KS17, which is not affected by RIP. The graph was created in The RIPper online software tool [66] (https://theripper.hawk.rock (accessed on 25 February 2022).

Analysis with REPET showed that the 7 Kbp region of *F. circinatum* FSP34 (i.e., the stretch of DNA lacking the two genes present in *F. temperatum*) contains three unclassified Large Retrotransposon Derivatives (RXX-LARD), which together span 5926 bp (Table S5 in Supplementary File S4). BLAST analyses with these TEs showed that none of them matched other sequences in the genome of *F. circinatum* FSP34. For one of them, however, regions with >75% sequence similarity were detected at two positions on chromosome 2 of *F. circinatum* KS17 and at two positions on chromosome 11 of *F. temperatum* (Table S6 in Supplementary File S4). Furthermore, as expected for TE-rich regions, we detected evidence of RIP in the ca. 7000 bp of *F. circinatum* FSP34 (Figure 5). RIP index values indicative of RIP was recorded from 1,120,500 bp to 1,127,500 bp on chromosome 11 of *F. circinatum* FSP34, while the corresponding region in *F. circinatum* KS17 and *F. temperatum* appeared not to have been affected by RIP (Figure 5). 

### 3.4. Growth and Distribution of QTL Genes in Other F. circinatum Isolates

We used a PCR approach to determine the presence of *F. temperatum* genes g13407 and g13408 in the genomes of genetically diverse individuals of *F. circinatum* (Table 2). In most cases (and in *F. temperatum* and *F. circinatum* KS17 serving as positive controls), amplicons of approximately 1002 bp for gene g13407 and 670 bp for gene g13408 were generated. These had the same size and sequence as what was predicted from the genomes of *F. temperatum* and *F. circinatum* KS17. However, similar to what was found for isolate FSP34 of *F. circinatum*, no amplicons were generated for either of the two genes in four *F. circinatum* isolates (CMWF559, CMWF508, CMWF509, and CMWF 528). Overall, the isolates originating from Mexico and South Africa all contained both genes, while it was absent from some isolates originating from Florida and California. 

We also used a PCR approach to investigate the presence/absence of gene g13376, located 17 Kbp downstream of the gene closest to the estimated position of the QTL in *F. circinatum* FSP34, as well as the gene occupying the same position in KS17 (g13276). For all the *F. circinatum* isolates tested, we recovered amplicons of either 2388 bp (similar to that of *F. circinatum* FSP34) or 2229 bp (similar to *F. circinatum* KS17). None of the individuals tested encoded the *F. temperatum* gene found at this genomic location. However, the distribution patterns of these genes were different from those observed for g13407 and g13408 (i.e., the two missing in *F. circinatum* FSP34 but present in *F. temperatum* and *F. circinatum* KS17). 

No pattern was apparent in the relative growth rates of the respective *F. circinatum* isolates and whether they contained genes g13407 and g13408 or not. The same was also true for the FSP34 gene g13376 and KS17 g13276 (Table 2). For example, isolate FSP34 grew significantly faster than *F. circinatum* KS17 (*p* = 6.67 × 10^−3^), but they both grew significantly (*p* = 1.09 × 10^−11^ and *p* = 6 × 10^−7^, respectively) faster than *F. temperatum* (Figure 1). However, the four isolates lacking genes g13407 and g13408 grew significantly slower than both isolates FSP34 and KS17 of *F. circinatum* (*p* < 0.055). In addition, some of the isolates (i.e., CMWF523, CMWF1802, and CMWF41612) that contained these genes also grew significantly slower (*p* < 0.05) than *F. circinatum* FSP34 and KS17, while others did not differ significantly from these two isolates (*p* > 0.05). 

## 4. Discussion

In this study, the mycelial growth rate of *F. circinatum* FSP34 and *F. temperatum* was investigated with the intention of identifying and characterizing QTLs linked to growth on pine-tissue-derived growth medium. One significant QTL (*p* < 0.05) was identified and localized to the genomes of *F. circinatum* FSP34 and *F. temperatum,* as well as several other sequenced members of the FFSC. A comparison of this QTL region in all investigated members of the FFSC revealed two genes, spanning 7090 bp, that were absent from the genome of *F. circinatum* FSP34. Instead, this region in *F. circinatum* FSP34 was characterized by low G+C content, the presence of LARD-type TEs, and nucleotide substitution indices suggestive of RIP. This QTL accounted for a large proportion (16.4%) of the total phenotypic variation for the significantly faster growth rate of *F. circinatum* FSP34 on pine-tissue-derived media, observed in this study.

Heritability values as large as that observed for growth on PMEA have been observed previously in other studies [27,72,73], suggesting a minimal influence of external environmental factors on the observed trait. Nevertheless, only one significant QTL was detected in this study. This could be explained by the complexity of growth as a trait [29]. In other words, the trait investigated here may be under the control of numerous loci that are additive to the manifestation of the trait observed [74,75,76]. Therefore, only one QTL which has a significant effect was able to be detected. Additionally, our QTL was not similar to QTLs detected in a previous study and did not occur on the same chromosome as those found to be involved in the growth of *F. circinatum* on potato dextrose agar [27].

Despite the high levels of genomic collinearity and synteny typically observed in the FFSC, our study suggests that intra-specific exceptions might be common and biologically informative. The conservation of gene content and orientation in the identified QTL region mirrored what was seen in the corresponding region of various other members of the FFSC examined (i.e., *F. verticillioides*, *F. subglutinans*, *F. fujikuroi*, *F. pilosicola*, and *F. fracticaudum*) [12,13,69,70,71], which is congruent with the macrosyntenic nature of the genomes of these fungi [13,16,77]. However, the QTL region in isolate FSP34 of *F. circinatum* lacked two genes that were present in all other isolates of this species, for which genome data is available [12,13,56,69]. This was further supported by the break in alignment observed in the MUMmer plots when comparing the *F. circinatum* FSP34 region to another strain of *F. circinatum* (KS17) [28] and to *F. temperatum* (Netza9) [56]. Our analysis also revealed a second striking difference between the genomes of these two fungi, where genes at the same location encoded different protein products. Therefore, in addition to providing a detailed illustration of gene content differences among strains of *F. circinatum*, this study reports a situation where the absence of genes positively influences a trait only in some isolates of the species. We hypothesize that the genic peculiarities underlying the QTL in FSP34 of *F. circinatum* are responsible for enhanced growth and not necessarily survival under the conditions examined. 

The two genes that were absent in the QTL region of *F. circinatum* FSP34 respectively encode a membrane protein SUR7/RIM9-like protein and a gene containing a flavin adenine dinucleotide (FAD)-type 2 binding domain. A direct link to growth has not been reported for the latter protein family, but it has been shown essential for assisting FAD to bind to metabolites such as fatty acids for their oxidation and hydroxylation [78]. With regards to the SUR7/RIM9-like membrane protein, RIM proteins form part of the SUR7 protein family [79], which contains two classes based on the number of amino acids that they encode [79]. Class 1 are short proteins (RIM9-like proteins) consisting of 225–399 amino acid residues, while Class 2 proteins are larger (PalI-like proteins) containing 476–756 amino acid residues. Both these classes of protein can occur in the same species (e.g., *Saccharomyces cerevisiae* and *Candida albicans*) [79,80], but a fungus may also only encode one. For example, *Aspergillus nidulans* only encode the PalI protein [79], as is the case in *F. circinatum* FSP34. Both these classes of proteins have been postulated and shown to be involved in response to various stresses through the RIM101/PalC pathway [79,81,82]. These stresses include alkaline pH response, nutrient limitation, and temperature [83,84]. Although no direct link between the presence/absence of RIM9-like proteins and increased growth have been recorded, knockouts of the RIM101 pathway have been shown to cause slow growth at a lower temperature in the yeast *S. cerevisiae* [83] and to be involved in growth development and stress response of *Trichothecium roseum* [85]. 

The polymorphic gene located 17 Kbp downstream of the two-gene deletion in *F. circinatum* FSP34 and its counterpart in *F. circinatum* KS17 encode different protein products, both lacking any known protein domains. Their counterpart in *F. temperatum* codes for a CorA Mg^2+^ transporter protein, which in fungi is known to be important for Mg^2+^ transport across the plasma membrane [86]. Magnesium is a well-known important cofactor for numerous biological processes such as DNA repair, neutralizing of charged phosphates, stabilization of the cell wall, and regulating electrolyte transport through the cell membrane, all of which are processes that may be implicated in growth [87,88]. As with the genes encoding SUR7/RIM9-like and FAD-type 2 binding domain proteins, the gene encoding the CorA Mg^2+^ transporter protein and its two counterparts in *F. circinatum* FSP34 and KS17 would require detailed gene knockout and replacement studies to fully understand their role in determining fungal growth (if any) or any other biological functions linked to them. The latter is important as genes underpinning QTLs may be pleiotropic [89], thus being involved in multiple biological pathways linked to fungal growth. Such as in the case of the CorA Mg^2+^ transporter protein, where through its function, it affects many processes [87,88].

The overall trend of harboring whole-gene polymorphisms at the two positions underlying the QTL in *F. circinatum* FSP34 was also evident when we examined a broader set of 19 *F. circinatum* strains. At the one locus, some strains harbored the two-gene deletion, and other isolates contained the genes, while at the second locus, some strains encoded the FSP34 version of the gene, and others encoded the KS17 gene. There was, however, not a clear correlation between the respective distribution patterns nor a clear association with growth rate in culture. This is likely due to the QTL accounting for only 16.4% of the variation observed and growth is a complex trait determined by numerous loci. Moreover, despite the fact that such large-effect QTLs as the one identified here are easier to detect as compared to small-effect QTLs [76,90], they often consist of multiple small-effect QTLs [91,92]. Furthermore, QTLs associated with complex traits may have context-dependent effects by being dependent on factors such as the prevailing environment, mode of reproduction, and genetic background [89]. Thus, the effect of this QTL might differ or not be observed in isolates of different genetic backgrounds or when other environmental conditions are imposed. Therefore, to fully understand the effects of the QTL detected in this study, future work focussed on studying growth under different environmental conditions and incorporation of qualitative information on variations observed in proteins or metabolites involved in growth may provide more insight into the growth of *F. circinatum* and the possible pleiotropic nature of QTLs associated with growth. 

Analysis of TEs suggested an origin involving retrotransposons for the region located within the QTL region of *F. circinatum* FSP34. It contained three unclassified Large Retrotransposon Derivatives (LARDs), which are a type of non-autonomous retroelement that are dependent on other TEs for their transposition (i.e., transcription, reverse transcription, and integration of the cDNA copies) [93,94]. Such as other TEs, LARDs are known to have an impact on the organization of a genome [95] through their insertion near or within a gene, which results in a negative impact on gene function, chromosomal rearrangements, and potentially deleterious effects [95,96]. The presence of at least three LARDs in the indel region of *F. circinatum* FSP34 thus points toward a possible role in the initial deletion of the two genes from the QTL region of this strain. 

A practical consequence of the presence of LARDs in the QTL region of *F. circinatum* FSP34, combined with its AT-richness, is that the predicted size of the region in the two-gene indel region could be significantly larger than what was observed in the current assembly of the isolate, despite the use of the longer read MinION technology. It is well-known that some genome assembly programs struggle with the assembly of AT-rich regions [67]. As a consequence, high similarity in tandem repeats, which the assembler programs read as similar, are concatenated. This makes it virtually impossible to identify the correct copy number of repeats in a region and causes the ultimate assembly to be smaller than its actual size [68]. This is likely why we were not able to amplify the DNA by PCR across the two-gene deletion region of isolate FSP34. Moreover, the presence of the ca. 7 Kbp region would not have been evident in the initial GLM as recombination frequency at this region did not contain the relevant AFLP markers [26]. Therefore, the identified stretch of DNA lacking the two genes that are present in *F. circinatum* isolate KS17 and in *F. temperatum* can thus be expected to be much larger in isolate FSP34 [89]. 

In this study, we were able to identify and characterize a QTL involved in the growth of *F. circinatum* FSP34 on PMEA. In addition, the superior growth of *F. circinatum* FSP34 has been putatively implicated to be linked to the absence of two genes within the genomic region encompassed by the QTL, which is being reported for the first time. This study has also identified TEs that may have aided in the deletion of these two genes in *F. circinatum* FSP34. These results provide valuable information in understanding the genetic basis of the growth of *F. circinatum* on host-derived media and are critical in understanding this fungal pathogen and its growth. The available resources, such as the GLM [26], the genomes [56,59], and the consequent study that has been conducted on the growth of *F. circinatum* [28], forms the backbone of understanding the role of specific genes involved in the growth of *F. circinatum*, and between different isolates of *F. circinatum,* as displayed in this study. Although QTLs in *F. circinatum* have been identified and characterized previously [27,28], and in this study, future research will involve examining the effects of the unique genes absent or present through knockouts and insertions and furthermore look at the effects on the closely related species *F. temperatum*, and other species within the FFSC and the *Fusarium* genus.

## Figures and Tables

**Table 1:** *Fusarium circinatum* isolates used in this Study.

Isolate Number *	Vegetative Compatibility Group (VCG)	Origin	References
CMWF543	12	South Africa	[48,49,50]
CMWF545	9	South Africa	
CMWF656	7	South Africa	
CMWF513	11	South Africa	
CMWF674; KS17	-	South Africa	
CMWF1799	A1	Mexico	
CMWF1800	A2	Mexico	
CMWF1801	A3	Mexico	[50,51]
CMWF1802	A4	Mexico	
CMWF1803	A5	Mexico	
CMWF531	C8	California	[51]
CMWF549	C6	California	
CMWF559	C2	California	
CMWF507	C3	California	
CMWF350; FSP34	C1	California	
CMWF508	SE34	Florida	
CMWF509	SE35	Florida	
CMWF523	SE32	Florida	
CMWF528	SE41	Florida	
CMWF529	SE40	Florida	

* CMWF/CMW = cultures obtainable from the Culture Collection based at the Forestry and Agricultural Biotechnology Institute (FABI), University of Pretoria, South Africa.

**Table 2:** Mycelial growth of genetically and geographically diverse isolates of *F. circinatum* on pine-based growth medium ^1^.

Isolate Number ^2^	Country	Mean Growth	±Standard Deviation	Tukey HSD ^3^
CMWF513 *^k^*	South Africa	55.5	0.35	hi
CMWF545 *^k^*		54.8	1.13	f
CMWF656		49.5	0.68	h
CMWF543		52.8	0.94	fg
CMWF674 (KS17) *^k^*		58.9	1.80	cd
CMWF508 *	Florida	42.7	1.00	j
CMWF528 *^k^		50.0	0.24	gh
CMWF523		46.3	0.47	i
CMWF529		60.5	0.54	bcd
CMWF509 *		55.6	0.89	ef
CMWF1802 *^k^*	Mexico	48.8	0.39	hi
CMWF1799 *^k^*		55.3	1.87	ef
CMWF1800		62.4	0.98	ab
CMWF1801		61.9	0.55	abc
CMWF1803 *^k^*		59.4	0.42	bcd
CMWF559 *	California	54.3	1.13	f
CMWF507		50.8	0.52	gh
CMWF531		61.3	1.45	abc
CMWF549		58.0	0.41	de
CMWF350 (FSP34) *		64.2	1.15	a
CMWF41612		39.9	1.04	j

^1^ Growth in mm after 7 days of incubation on PMEA (pine-tissue extract containing malt extract agar) medium. ^2^ Isolates lacking the 7Kb region are indicated with an asterisk (*). Isolates encoding the KS17 version of gene g13365 (and not the gene found at the same position in FSP34) are indicated with ^*k*^. ^3^ Isolates with the same letter are not statistically different from one another following Tukey’s test of the combined means (*p* < 0.05).

## Data Availability

Not applicable.

## Supplementary Materials

The following supporting information can be downloaded at: https://www.mdpi.com/article/10.3390/jof8111214/s1, Supplementary File S1: HPLC and GC-MS results showing the broad overview and primary metabolites identified in the pine-based media. Supplementary File S2: The primer sequences and PCR protocols used to amplify gene regions in this study. Supplementary File S3: Reference mapping of *F. circinatum* Illumina and MinIon raw reads mapped to the genomes of *F circinatum* KS17 and *F. temperatum*. Supplementary File S4: Genic information of the identified genes and indel region in the QTL region of *F. circinatum.* InterProScan and gene ontology information are provided for all genes in this region. Further information on the retrotransposons and repeats that are characteristic of the indel within the QTL region is provided.

## Design of the Study

In the collection, analyses, or interpretation of data, in the writing of the manuscript, or in the decision to publish the results.
