# Supplementary material for: Identification and Characterization of a QTL for Growth of Fusarium circinatum on Pine-Based Medium
Source: J Fungi (Basel). 2022 Nov 16;8(11):1214. doi: 10.3390/jof8111214 (PMC9696871; doi:10.3390/jof8111214)
Supplement: Supplementary file 1 [file jof-08-01214-s001.zip › Supplementary file S1.pdf]

Identification and characterization of a QTL for growth of *Fusarium circinatum* on pine-based medium

Benedicta S. Swalarsk-Parry[1], E.T. Steenkamp[1], S. van Wyk[1], Q.C. Santana[1,2], M.A. van der Nest[1,2], A. Hammerbacher[3], B.D. Wingfield[1] and L. De Vos[1]

[1] Department of Biochemistry, Genetics and Microbiology, Forestry and Agricultural Biotechnology Institute (FABI), University of Pretoria, Pretoria 0002, South Africa.
[2]Agricultural Research Council, Biotechnology Platform, 100 Old Soutpan Road, Onderstepoort, Pretoria, South Africa.
[3] Department of Zoology and Entomology, Forestry and Agricultural Biotechnology Institute (FABI), University of Pretoria, Pretoria 0002, South Africa.

Supplementary File S1

To obtain a broad overview of the metabolites conferred by the pine extract to the PMEA, High-Performance Liquid Chromatography (HPLC) were used according to Hammerbacher et al. (2014). The solidified media (i.e., containing pine extract and agar, but lacking malt extract) were separated on a Nucleodur Sphinx PR18ec column (Macherey-Nagel) by utilizing a flow rate of 1.0 ml.min-1 on the Agilent 1100 series HPLC. The temperature of this column was kept constant at 25°C. To separate the phenolic compounds, 0.2% of formic acid and acetonitrile were used as the mobile phases. Furthermore, to quantify and identify the main compound groups, the Esquire 6000 ESI-ion trap mass spectrophotometer was utilized. These analyses showed that the medium had three main compound groups, namely primary metabolites, lignin-derived secondary metabolites, and flavonoid derived secondary metabolites (see figure below).

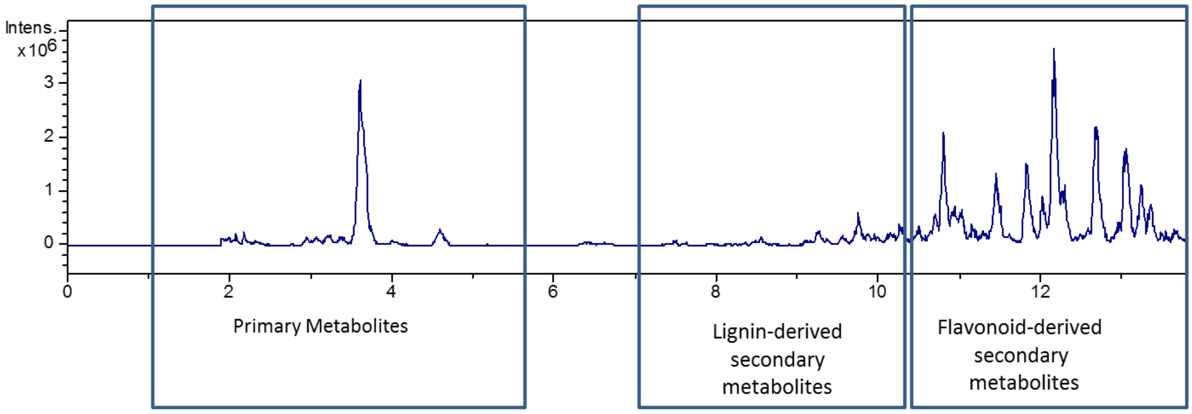

HPLC results show the broad groups of metabolites detected in pine-based media. The Y-axis indicates the intensity of the peaks and the X-axis indicates time (min).

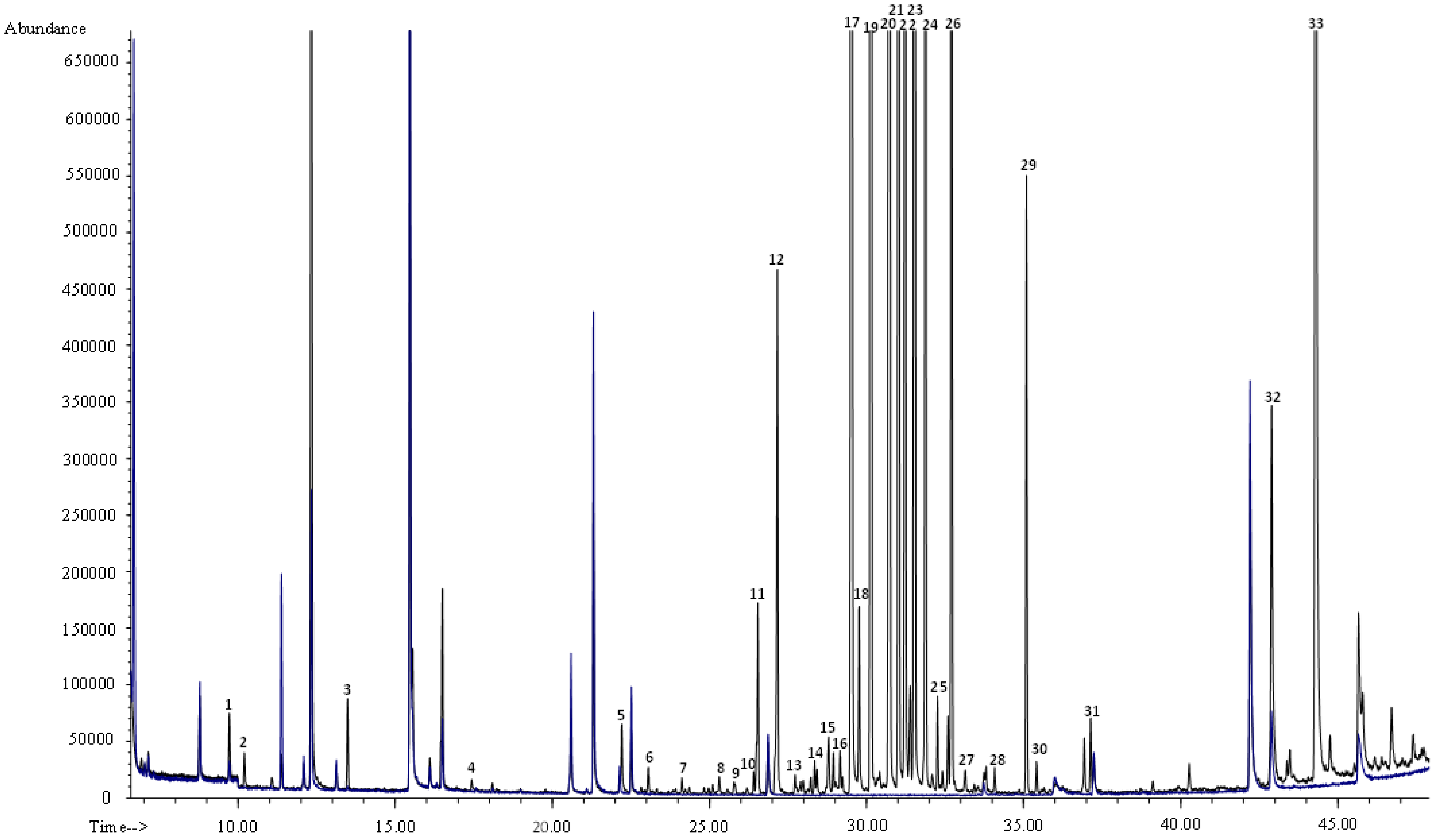

GC-MS chromatogram to show the primary metabolites identified in the pine-based media. The Y-axis indicates the abundance of the metabolites, and the X-axis indicates time (min).

Primary metabolites identified using GC-MS in Figure 2.	
1. Acetic acid	18. Deoxy-pentaric acid
2. Alanine	19. Myo-Inositol
3. Hydroxy-methoxy-benzoic acid	20. Unidentified diterpenoid
4. Succinate	21. Fructose
5. Malate	22. Fructose
6. Butanate	23. Glucose
7. Trihydroxybuturic acid	24. Mannose
8. Pentose sugar	25. Talose
9. Trimethoxybenzene	26. Myo-Inositol
10. Xylose	27. Glucose
11. Ribose	28. Hexose sugar
12. Galactose	29. Inositol derivative
30. Pentose sugar	30. Inositol derivative
31. Glucuronic acid	31. Hexose sugar
3. Ribonic acid	32. Hexadecanoic acid
33. Ribonic acid	33. Sucrose
34. Deoxy-mannose	
