# Supplementary material for: Identification and Characterization of a QTL for Growth of Fusarium circinatum on Pine-Based Medium
Source: J Fungi (Basel). 2022 Nov 16;8(11):1214. doi: 10.3390/jof8111214 (PMC9696871; doi:10.3390/jof8111214)
Supplement: Supplementary file 1 [file jof-08-01214-s001.zip › Supplementary file S2.pdf]

Identification and characterization of a QTL for growth of *Fusarium circinatum* on pine-based medium

Benedicta S. Swalarsk-Parry[1], E.T. Steenkamp[1], S. van Wyk[1], Q.C. Santana[1,2], M.A. van der Nest[1,2], A. Hammerbacher[3], B.D. Wingfield[1] and L. De Vos[1]

[1] Department of Biochemistry, Genetics and Microbiology, Forestry and Agricultural Biotechnology Institute (FABI), University of Pretoria, Pretoria 0002, South Africa.
[2]Agricultural Research Council, Biotechnology Platform, 100 Old Soutpan Road, Onderstepoort, Pretoria, South Africa.
[3] Department of Zoology and Entomology, Forestry and Agricultural Biotechnology Institute (FABI), University of Pretoria, Pretoria 0002, South Africa.

Supplementary File S2

The sequence of the primers used to confirm the absence or presence of genes and regions targeted in this study.

Primer name	Primer sequence (5' to 3')	Target gene	Target region
P_{73F}	ACTCCCCAACTGACTCATGG	g13407	1 194 142 - 1 195 143
P_{73R}	CCACTCGCTTCCAAACACAA		
P_{74F}	CAGTTGGCACTAGTCCTCCA	g13408	1 198 252 - 1 198 921
P_{74R}	TCAGGATCGATACCAAGGGC		
P_{4F}	TTGCCCTCTCCAGTACCAAG	FSP34 indel	1 119 532 - 1 127 164
P_{5R}	TGTCTTTGGTTCACACGCTG		
$KS17_F$	GATGACAAACCGGCTCACTC	g13276	1 206 036 - 1 208 265
$KS17_R$	CAGCCATGAGACGGTAAAGC		
$FSP34_F$	CATGGACGCGTCAAGTACTG	g13376	1 165 752 - 1 168 139
$FSP34_R$	TGCTTGCAGGTTCAAAGGTC		
FT_F	GGTGGTGCTAAGGACTCACT	g13420	1 238 853 - 1 242 201
FT_R	GGTGCTCGCTTTCCAATAGG		

Cycling conditions using the KAPA *Taq* PCR kit (Kapa Biosystems) – these conditions were mainly used for the genes g13407 and g13408

Step	Temperature	Time	Cycles
Initial Denaturation	95 °C	3 min	1
Denaturation	95 °C	30 seconds	30
Annealing	variable *	30 seconds	
Extension	72 °C	1 min per kb	
Final Extension	72 °C	5 minutes	1
Hold	4 °C	∞	

*Annealing temperature for primer set P_{73F+R} = 57 °C primer set P_{74F+R} = 56 °C

**Cycling conditions using the LongAmp® *Taq* 2X Master Mix (New England BioLabs) – these conditions were mainly used for the indel in *F. circinatum* FSP34**			
**Step**	**Temperature**	**Time**	**Cycles**
Initial Denaturation	94 °C	30 seconds	1
Denaturation	94 °C	30 seconds	
Annealing	55 °C	1 minute	10
Extension	65 °C	8 minutes	
Denaturation	94 °C	30 seconds	
Annealing	55 °C	1 minute	20
Extension	65 °C	8 minutes + 20 sec each cycle	
Final Extension	65 °C	10 minutes	1
Hold	4 °C	∞	

**LongAmp® *Taq* 2X Master Mix (New England BioLabs) protocol to amplify three different genes* in CMWF350 (FSP34), CMWF389, and CMWF674 (KS17).**			
**Step**	**Temperature**	**Time**	**Cycles**
Initial Denaturation	94 °C	30 seconds	1
Denaturation	94 °C	30 seconds	
Annealing	52 °C	30 seconds	30
Extension	65 °C	2 minutes 30 seconds	
Final Extension	65 °C	10 minutes	1
Hold	4 °C	∞	

*Primer set $FSP34_{F+R}$, $KS17_{F+R}$, and FT_{F+R} were used to amplify the different genes in *F. circinatum* (FSP34 and KS17) and *F. temperatum* (CMWF389).
