# Supplementary material for: Identification and Characterization of a QTL for Growth of Fusarium circinatum on Pine-Based Medium
Source: J Fungi (Basel). 2022 Nov 16;8(11):1214. doi: 10.3390/jof8111214 (PMC9696871; doi:10.3390/jof8111214)
Supplement: Supplementary file 1 [file jof-08-01214-s001.zip › Supplementary file S3.pdf]

Identification and characterization of a QTL for growth of *Fusarium circinatum* on pine-based medium

Benedicta S. Swalarsk-Parry[1], E.T. Steenkamp[1], S. van Wyk[1], Q.C. Santana[1,2], M.A. van der Nest[1,2], A. Hammerbacher[3], B.D. Wingfield[1] and L. De Vos[1]

[1] Department of Biochemistry, Genetics and Microbiology, Forestry and Agricultural Biotechnology Institute (FABI), University of Pretoria, Pretoria 0002, South Africa.
[2]Agricultural Research Council, Biotechnology Platform, 100 Old Soutpan Road, Onderstepoort, Pretoria, South Africa.
[3] Department of Zoology and Entomology, Forestry and Agricultural Biotechnology Institute (FABI), University of Pretoria, Pretoria 0002, South Africa.

Supplementary File S3

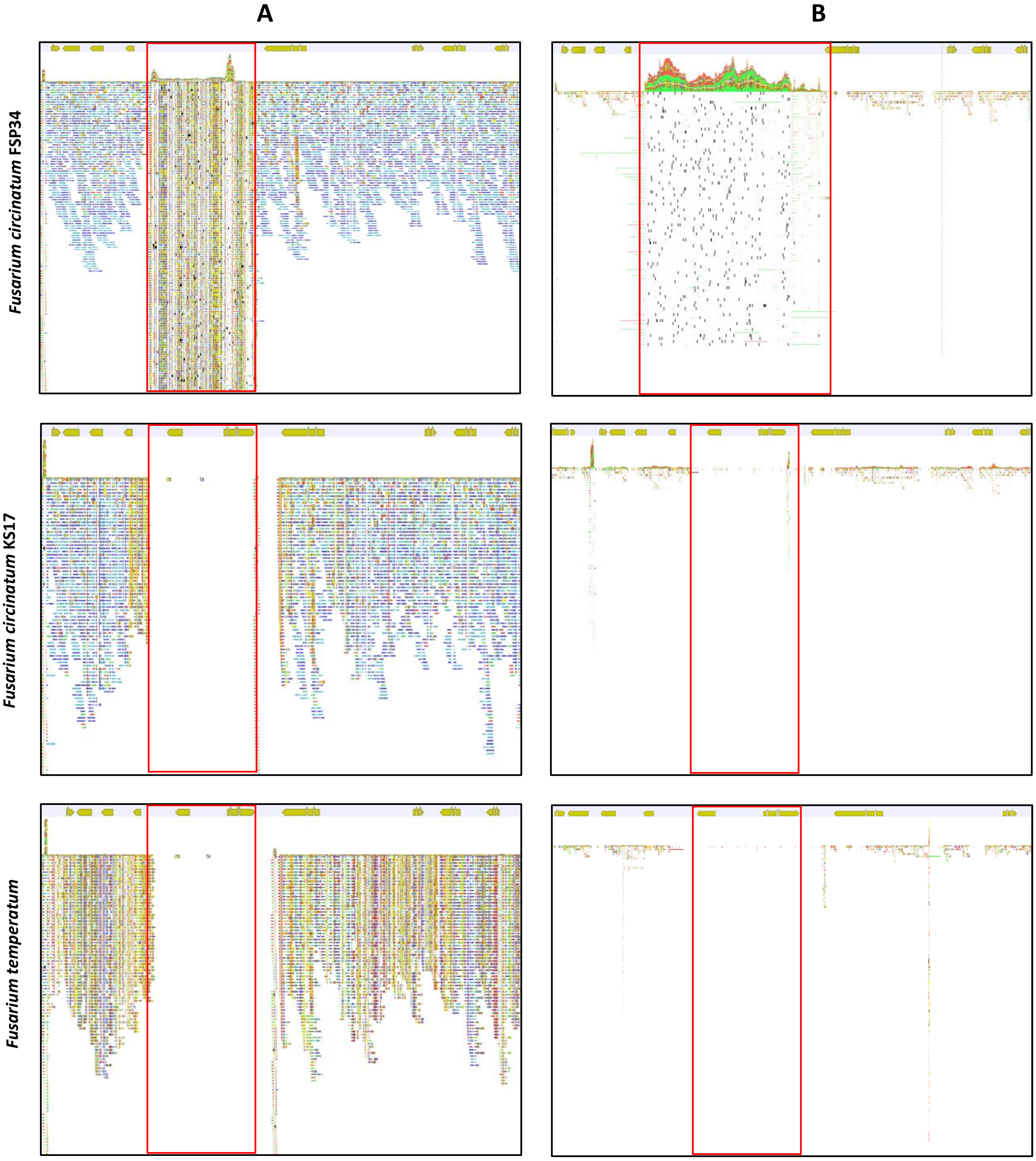

Reference mapping of *F. circinatum* (A) Illumina and (B) MinIon raw reads mapped to the genomes of *F. circinatum* (FSP34 & KS17) and *F. temperatum.* The red block indicates the location of the two gene indel and the coverage of the mapped reads.
